# Comparative Studies on Carbon Paste Electrode Modified with Electroactive Polyamic Acid and Corresponding Polyimide without/with Attached Sulfonated Group for Electrochemical Sensing of Ascorbic Acid

**DOI:** 10.3390/polym14173487

**Published:** 2022-08-25

**Authors:** Jiunn-Jer Hwang, Aamna Bibi, Yu-Ci Chen, Kun-Hao Luo, Hsiang-Yuan Huang, Jui-Ming Yeh

**Affiliations:** 1 Department of Chemical Engineeing, Army Academy, Taoyuan 32023, Taiwan; 2 Department of Chemistry and Center for Nanotechnology, Chung Yuan Christian University, Taoyuan 32023, Taiwan

**Keywords:** sulfonated group, electroactive, polyamic acid, polyimide, electrochemical sensor, ascorbic acid

## Abstract

In this study, electroactive poly (amic acid) (EPAA) and corresponding polyimide (EPI) without or with a sulfonated group (i.e., S-EPAA, and S-EPI) were prepared and applied in electrochemical sensing of ascorbic acid (AA). The electroactive polymers (EAPs) containing EPAA/EPI and S-EPAA/S-EPI were synthesized by using an amine-capped aniline trimer (ACAT) and sulfonated amine-capped aniline trimer (S-ACAT) as an electroactive segment that controlled the redox capability and influenced the degree of sensitivity of the EAPs towards AA. Characterization of the as-prepared EAPs was identified by FTIR spectra. The redox capability of the EAPs was investigated by electrochemical cyclic voltammetric studies. It should be noted that the redox capability of the EAPs was found to show the following trend: S-EPAA > S-EPI > EPAA > EPI. For the electrochemical sensing studies, a sensor constructed from an S-EPAA-modified carbon paste electrode (CPE) demonstrated 2-fold, 1.27-fold, and 1.35-fold higher electro-catalytic activity towards the oxidation of AA, compared to those constructed using a bare CPE, S-EPI-, and EPI/EPAA-modified CPE, respectively. The higher redox capability of S-EPAA-modified CPE exhibited a good electrochemical response towards AA at a low oxidative potential, with good stability and selectivity. Moreover, an electrochemical sensor constructed from S-EPAA-modified CPE was found to reveal better selectivity for a tertiary mixture of AA/DA/UA, as compared to that of EPI-modified, EPAA-modified and S-EPI-modified CPE, based on a series of differential pulse voltammograms.

## 1. Introduction

Vitamin C plays a vital role in the production of neurotransmitters and tissue repair [1]. It is necessary for the functions of several enzymes and the regulation of various biological functions, including functioning of the immune system, wound healing and collagen synthesis [2]. It is an essential nutrient for higher primates and a few other organisms. Vitamin C deficiency can cause scurvy [3,4]. The pharmacodynamic group of vitamin C is ascorbate ion. It is also of prime importance in some metabolic redox reactions in the human body. Therefore, ascorbic acid (AA) testing is of prime importance in the food, pharmaceutical and clinical industries. A variety of techniques for AA determination, including spectroscopy, chromatography, enzymatic and electroanalysis, have been reported [5,6,7,8]. Among them, because of its high sensitivity and simple operation, the electrochemical method is considered one of the best potential methods.

The sensitive materials currently used in electrochemical detection contain inorganic or organic materials. Studies have found that inorganic materials have excellent sensing effects but require a higher operating temperature to absorb and release samples. Therefore, organic sensing materials such as the conducting polymers had been utilized by various groups in the development of sensors. Among the conductive polymers, polyaniline (PANI) has a low cost, good environmental stability and unique doping/de-doping and reversible redox capabilities. In 1997, researchers [9] used the electro-deposition method with pol(3-methylthiophene), polypyrrole (PPy) and PANI, which were modified on platinum conductive electrodes, to detect different organic and biological molecules (such as vitamin C); they found that different conductive polymers can enhance the ability to sense vitamin C because of their electroactivity. Sensing ascorbic acid using an electroactive polymer (EAP) containing aniline has been practiced since 2007. Battaglini et al. used aniline and *N*-(3-propane sulfonated) aniline (PSA) as EAPs by electrochemical polymerization. They found that the modification of PSA and aniline with a synthesis ratio of 1:9 on the glassy-carbon electrode surface can obtain the highest electroactivity [10]. Therefore, how to strengthen the electro-activity of the material has become the most critical factor for electrochemical sensors.

Several useful approaches have been employed to enhance the water solubility and maintain the electrochemical activity of PANI in a neutral pH, such as the development of self-doped PANI (SPANI). SPANI exhibits improved solubility, electrical properties and redox activity over a wide range of pH, along with a conductivity of 10^−6^–10^−1^ S/cm. Thus, it has promising applications in various fields, including rechargeable batteries [11], LEDs [12], cell scaffolds [13], junction devices [14], electrochromic devices [15] and biosensors [16]. SPANI can be formed through the introduction of SO_3_H, COOH, Ph-B(OH)_2_ and H_3_PO_3_ groups on the side chains or directly onto the aniline ring [17,18]. The introduction of the sulfonic acid group is a cost effective method and it undergoes self-doping via a stable six-membered ring structure with imine or amine nitrogen [19]. 

Shi et al. designed a simple and highly sensitive ammonia sensing device with a low detection of limit based on PPY/SPANI composite film [20]. In 2018, Li et al. used SPANI/chitosan and reported a remarkable amplification in the cathodic current response for the electrochemical sensing of chloramphenicol [21]. Zheng et al. developed a dopamine electrochemical sensor by combining carbon nano-spheres and SPANI in 2019 [22]. Subsequently, Fungaro used SPANI for the voltammetric detection of Zn, Pb and Cd in water [23]. Similarly, in 2010, Gopalan used SPANI-g-MWCNT for biosensing glucose [24]. Although the introduction of the SO_3_H group altered the various properties of PANI, the structural properties are yet to be fully investigated, because the synthesized PANI/SPANI consist of chain entanglement, coils, large less-ordered domains and polydispersity. Hence, oligomers with flexible side groups and defined molecular structures could help us understand molecular arrangements, which could provide attractive properties that can be fine-tuned for use in different applications [25].

In recent years, various articles have been published on oligo-aniline-based EAPs because of their excellent solvent solubility, mechanical strength and biodegradability. Other authors have reported a series of EAPs [26,27,28,29]. For instance, Yeh et al. synthesized EAPs based on aniline oligomers for the electrochemical sensing of AA [30,31,32,33,34,35]. In 2017, Yang et al. reported graphene oxide-based sulfonated oligo-aniline coatings for marine corrosion protection applications [36]. Joseph et al. used sulfonated aniline oligomers for the optical detection of hemoglobin. [37] In 2019, Yeh et al. reported a comparative study on a non-sulfonated amino-capped aniline trimer (ACAT) and sulfonated amino-capped aniline trimer (S-ACAT)-based electroactive polyurea as corrosion inhibitors [38]. 

Based on the previously published literature, EAPs without/with sulfonic acid group were prepared and applied in the electrochemical sensing of AA in this study. First of all, ACAT and S-ACAT were synthesized by oxidative coupling reactions. Subsequently, a series of EAPs containing EPAA, EPI, S-EPAA, and S-EPI were synthesized. The effects of the attached –COOH and –SO_3_H groups on the redox capability of some as-prepared EAPs were investigated by electrochemical cyclic voltammetry (CV) studies. We envisioned that EAPs with a carboxylic and sulfonic acid group might influence the corresponding redox capability and, therefore, affect the electrochemical sensing of AA. Hence, in this study, an electrochemical sensor was constructed by a carbon paste electrode (CPE) modified with EPAA, EPI, S-EPAA and S-EPI and applied in the detection of AA. The sensitivity, selectivity, and stability of CPE modified with four distinctive EPAs were investigated and compared systematically. 

## 2. Experimental Section

### 2.1. Chemicals

Aniline (99%, Alfa aesar, Lancashire, UK) distilled before use, ammonium peroxodisulfate (APS) (J. T. Baker, Randor, PA, USA)), N-methyl-2-pyrrolidone (uniregion bio-tech, Taoyuan, Taiwan), hydrochloric acid (HCl, 37%, Honey well Riedel-de Haen, New Taipei, Taiwan), ammonium hydroxide solution (NH_4_OH, 28%, Honey well Riedel-de Haen, New Taipei, Taiwan) and methanol (MeOH, Miaoli, Taiwan), dimethylacetamide (DMAc Duksan, 99%, New Taipei, Taiwan), (CH_3_CO)_2_O, Pyridine (J. T. Baker, 98.9% Randor, PA, USA), ODA (4,4-oxydianiline), BSAA (4,4′-(4,4′-isopropylidenediphenoxy)bis(phthalic anhydride) (Aldrich 97%, St. Louis, MO, USA) and 4,4′-diaminodiphenylamine sulfate hydrate (TCI, 98%, Tokyo, Japan)) were used as received without further purification.

### 2.2. Instrumentation 

MS (mass spectrometer, Bruker Daltonics IT, Hsinchu County, Taiwan), ^1^H-NMR (proton nuclear magnetic resonance spectroscopy, Bruker 300 spectrometer, Hsinchu County, Taiwan) and FTIR spectrometer (JASCO FT/IR-4100, Easton, PA, USA) were used for the characterization of the as-prepared material. UV–vis (JASCO V-750, Easton, PA, USA), cyclic voltammetry (CV AutoLab PGSTAT 204 (Ω Metrohm Autolab, KM Utrecht The Netherlands) and GPC (gel permeation chromatography, Waters-150 CV, MA, USA) were used to determine the morphology, redox ability and molecular weights of the samples. 

### 2.3. Synthesis of N,N′-Bis(4′-aminophenyl)−1,4-quinonenediimine (acat)/3,6-bis((4-aminophenyl)imino)cyclohexa-1,4-diene-1-slfonic Acid (S-ACAT)

**Scheme 1:**
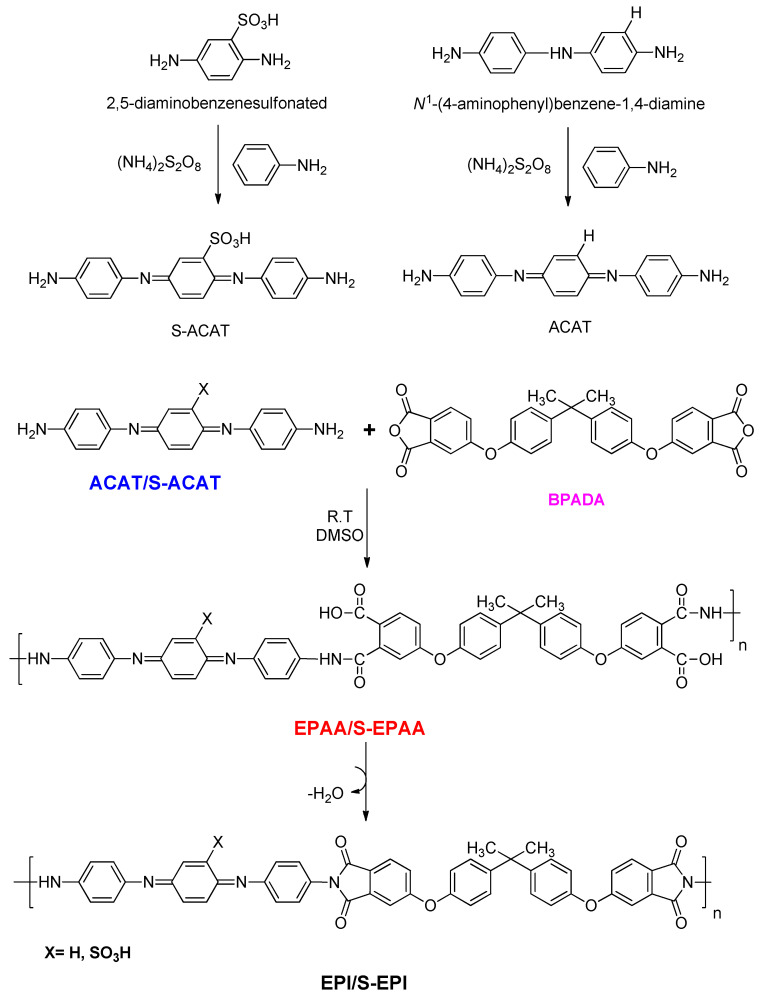
Preparation of EPAA/EPI and S-EPAA/S−EPI.

ACAT and S-ACAT were synthesized based on the procedure reported in the literature [38] (Scheme 1). The detailed procedure is given in the Supporting Information (SI).

### 2.4. Preparation of Electroactive Polyamic Acid (EPAA/S-EPAA) and Polyimide (EPI/S-EPI)

Electroactive polyamic acid and the corresponding polyimides were prepared as reported in the literature [39] (Scheme 1). Briefly, EPAA and S-EPAA were synthesized from the polymerization of BPADA and ACAT/S-ACAT, respectively. The respective monomer was dissolved in DMSO at room temperature under nitrogen atmosphere, and then BSAA was added slowly into the solution. After that, the mixture was stirred for 6 h followed by precipitation, filtration and vacuum drying in oven at 60 °C for 3 h to obtain EPAA and S-EPAA.

### 2.5. Redox Property of the as-Prepared Materials 

In this study, the synthesized materials (e.g., EPAA, S-EPAA, EPI and S-EPI) underwent CV measurements to determine the redox properties. Firstly, 1 wt% solution of the as-prepared materials was prepared in DMAc, followed by spin coating on ITO electrodes (5 × 5 cm^2^). Subsequently, the ITO electrodes were dried by heating at 120 °C for 1 h. CV measurements were recorded at room temperature, with a double-wall jacketed cell in 1.0 M H_2_SO_4_ with platinum foil and Ag/AgCl (3 M NaCl solution) served as the auxiliary and the reference electrode, respectively.

### 2.6. Preparation of Test Solutions

(1) PBS solution: 0.1 M PBS solution (pH 7.02) was prepared by dissolving 9.6 g of Dulbecco’s PBS powder in 1 L of deionized water.
(2) AA solution: 2 mM AA solution was prepared in PBS.
(3) CPE preparation: 30 mg carbon paste (weight ratio: graphite:paraffin oil, 3:1) and 20 mg of the given EAP were homogenously mixed and neatly filled in the groove at the front of the electrode to perform the electroactivity test.
(4) AA sensing: A homogeneous mixture of the as-prepared EAP (i.e., EPAA, S-EPAA, EPI and S-EPI) and carbon paste (60:6 mg) was prepared by grounding it with a mortar and pestle for the electrochemical sensing of AA. Subsequently, this mixture was packed into the Teflon tube (3 mm diameter) of the working electrode fitted with copper rod at the centre. Voltalab 50 and Autolab electrochemical workstations were used to perform all the voltammetric measurements in 30 mL of PBS (0.1 M). Platinum foil was used as the reference and Ag/AgCl (3 M NaCl solution) as the counter electrodes. The CPEs modified with EPAA, S-EPAA, EPI and S-EPI were used as the working electrodes at room temperature (25 °C).

## 3. Results and Discussion

### 3.1. Characterization 

**Figure 1:**
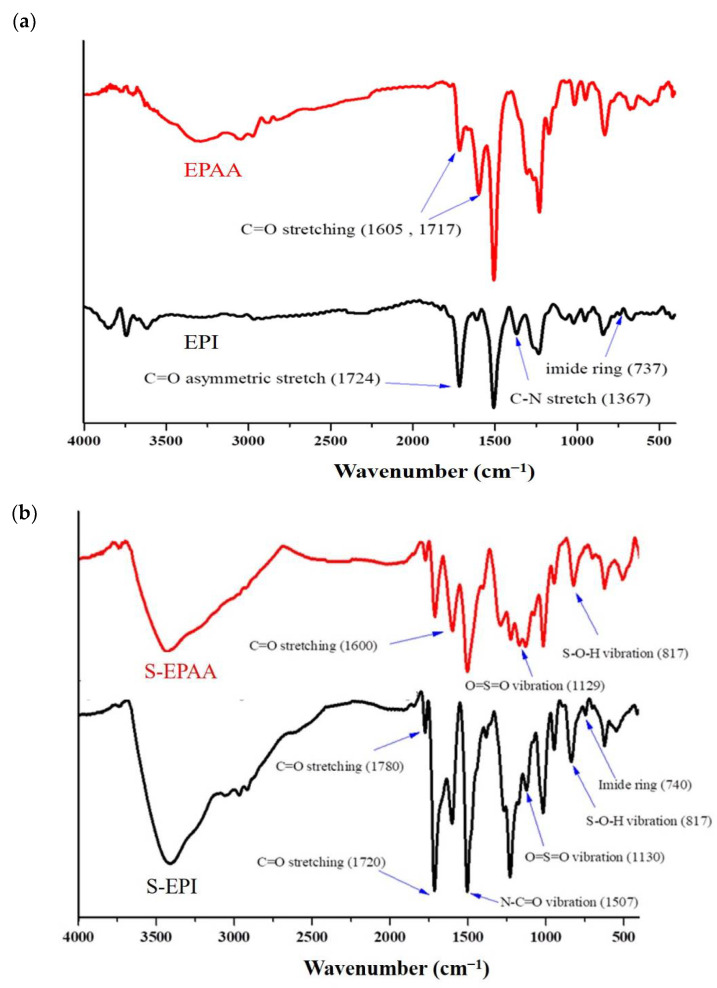
FTIR spectra of (**a**) EPAA and EPI; (**b**) S−EPAA and S−EPI.

ACAT and S-ACAT were fully characterized as shown in Figures S1 and S2, respectively. EPAA/S-EPAA and EPI/S-EPI were characterized by FTIR, as shown in Figure 1. The main vibration peaks in the spectrum of EPAA in Figure 1a are the stretching vibration of the amide group (C=O) at 1605 cm^−1^, carboxylic acid group (C=O) at 1717 cm^−1^ and the carboxylic acid group (O–H) at 2400–3400 cm^−1^. After the ring closure of EPI, the spectrum in Figure 1a shows that the original peaks at 1605 and 1717 cm^−1^ became a unique stretching band of the imide ring (C=O) at 1724 cm^−1^, and the vibration band of the carboxylic acid group (O–H) at 2400–3400 cm^−1^ also disappeared. In addition, the peak for imide ring (C–N–C) also appeared at 1367 cm^−1^ and the bending vibration of the imide ring (C–N–C) appeared at 740 cm^−1^. Similarly, in the FTIR spectrum of S-EPAA in Figure 1b, the original vibration peaks of the amide group (C=O) and carboxylic acid group (C=O) were observed at 1600 and 1715 cm^−1^, respectively. After the ring closure of the imidized material, the peak at 1720 cm^−1^ became remarkably enhanced in the FTIR spectrum of S-EPI, the stretching band of the imide ring (C–N–C) also appeared at 1370 cm^−1^, and the bending vibration of the imide ring (C–N–C) also appeared at 740 cm^−1^. The FTIR spectra confirmed that the four EAP materials, namely, EPAA, EPI, S-EPAA and S-EPI, were successfully prepared.

### 3.2. Polymer Electroactivity Measurement

**Figure 2:**
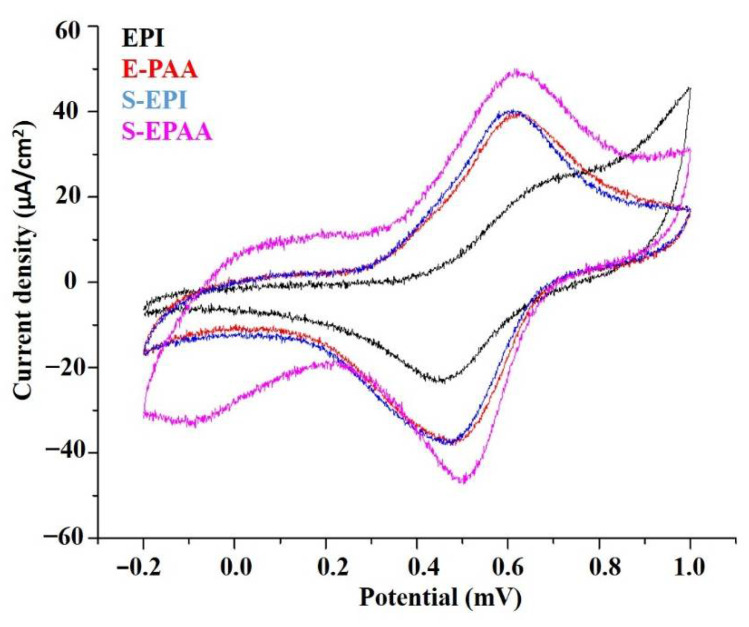
Electrochemical cyclic voltammetry for the as-measured samples in 1.0 M H_2_SO_4_ at a scan rate of 50 mV/s, with a potential range of −0.2 V~1.0 V.

The redox nature of ACAT/S-ACAT (Figure S3) and the as-synthesized samples (i.e., EPAA, EPI, S-EPAA and S-EPI) was tested by CV in 1 M H_2_SO_4_. The observed oxidation current density of EPAA (39 μA/cm^2^) was 1.6 times higher than that of EPI (24 μA/cm^2^). The results suggest that the two carboxylic acid groups of EPAA can result in a greater redox capacity. Using the above measurement method, the electroactive polyamic acid with sulfonic acid and the electroactive polyimide with sulfonic acid were compared. The results showed that the redox abilities of S-EPAA and S-EPI were greater than those of EPAA and EPI, respectively. S-EPI showed a current density of 40 μA/cm^2^, whereas the highest value observed for S-EPAA was a current density of 49 μA/cm^2^. The CV test result in Figure 2 shows that the redox capabilities of the EAPs were in the following order: SEPAA > SEPI > EPAA > EPI. The CV results also showed that EPAA/S-EPAA and EPI/S-EPI have reversible redox abilities. The principle of electrochemical catalysis can be used to oxidize AA in the oxidized aniline segment, and the current can be detected by CV to achieve the AA detection.

**Figure 3:**
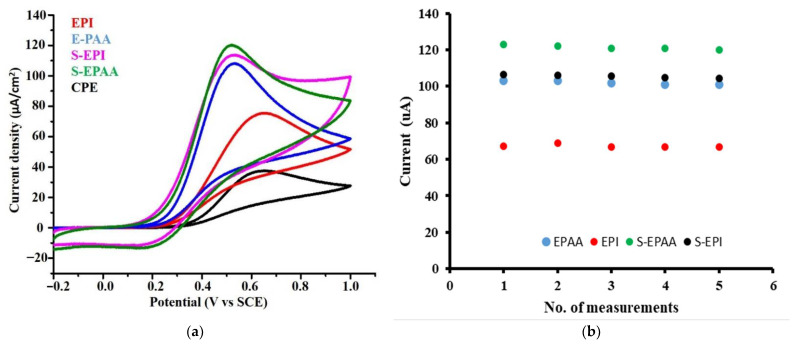
(**a**) Electrochemical cyclic voltammetry for the as-measured samples in 2 mM AA at a scan rate of 50 mV/s, with a potential range of −0.2 V~1.0 V. (**b**) The repeatability of the proposed sensor for five successive measurements in 200 μm AA.

Figure 3a shows the electroactivities of EPAA, EPI, S-EPAA and S-EPI via CV operating in 40 mL of 2 mM AA solution at 50 mV/s. The measured CV curves showed a clear oxidation peak, which indicates that AA was oxidized after electrochemical catalysis. The lower oxidation potential or the larger oxidation current, the more suitable the material is for the electrocatalytic oxidation of AA.

**Scheme 2:**
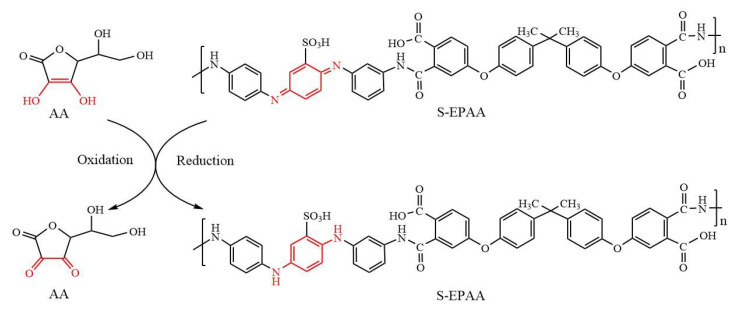
Redox reaction that occurred between AA and S-EPAA.

Oxidation currents were generated at the working electrodes of 2 mM AA in PBS solution. The electrode sensing ability of EPAA was better than that of EPI, and S-EPAA had better sensing ability than S-EPI. In general, the resonance effect of the sulfonate-enhanced aniline ring helps to enhance its oxidation ability, which results in better sensing ability for AA, and the best sensing ability was found in S-EPAA, which has sulfonate and carboxylic acid groups. Additionally, AA undergoes oxidation via the following mechanism, as shown in Scheme 2 [30].

Furthermore, the repeatability of the constructed electrochemical sensor for AA detection was also investigated, as shown in Figure 3b. It should be noted that, by detection of 2 mM of AA, S-EPAA-modified CPE was found to reveal good repeatability at the stable current density of 122 μA/cm^2^, with the minimum relative standard deviation of 0.43%, as shown in Figure 3b. However, the S-EPI-modified CPE was found to exhibit a stable current density of ~110 μA/cm^2^, with the minimum relative standard deviation of 0.59%. After this testing period, the constructed electrochemical sensor was found to retain ~93–94% of its initial response, indicating that the four constructed electrochemical sensors all reveal excellent repeatability.

### 3.3. Electrochemical Sensing of AA

#### 3.3.1. Amperometry

**Figure 4:**
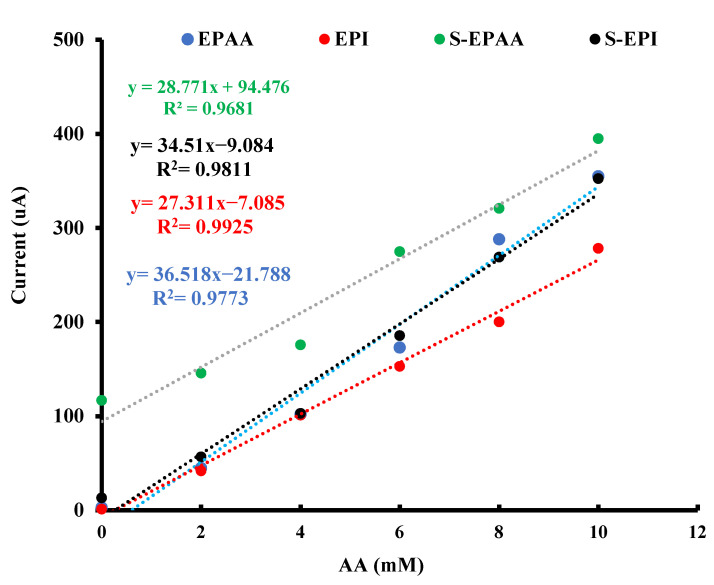
Linear calibration curves of CPE-modified EPAA, EPI, S-EPAA and S-EPI electrodes in PBS (pH 7) with different AA concentrations.

The cyclic voltammograms of the modified CPE electrodes performed at various concentrations of AA (0–10 mM) in PBS (pH 7.0) are shown in Figure S4. The detection was attributed to the reduction reaction of EPAA, EPI, S-EPAA and S-EPI and oxidation of AA. The increase in AA concentration increased the current value. Figure 4 shows the plot of the AA oxidation peak current density versus AA concentration, which indicates that the concentration of AA and oxidation peak current have a direct relationship and the slope values of 36.5, 27.3, 28.7 and 34.5 μA/mM were observed for EPAA, EPI, S-EPAA and S-EPI, respectively. These results indicate that the modified CPE electrodes effectively catalyzed the oxidation of AA.

**Figure 5:**
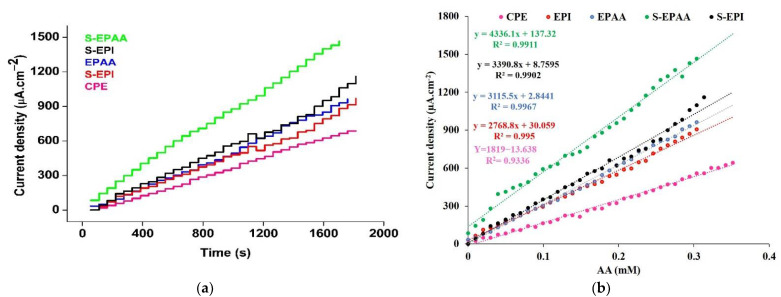
(**a**) Amperometric current responses of EPAA, EPI, S-EPAA and S-EPI-modified CPE electrodes for successive addition of AA into a 30 mL PBS that was stirred constantly. (**b**) Linear calibration curves between the current density and the concentration of AA.

Sensitivity is a very important reference value for materials used as sensors. The fixed-ampere method was used to test the current response ability and measure the difference in sensitivity between the CPEs and CPEs modified with EPAA, EPI, S-EPAA and S-EPI. The modified CPE was placed in a homogenized stirred solution of PBS (30 mL). The chronoamperometry program was set at 510 mV, with 5 s^−1^ scanning frequency. Figure 5a shows the amperometric responses of the bare CPEs and CPEs modified with EPAA, EPI, S-EPAA and S-EPI, with the continuous addition of AA in the PBS solution. The as-synthesized electrode increased the current successively with the successive addition of AA in 0.1 M PBS (potential 0.51 V). Each step showed a current increase with an increment of 15 mM AA. CPE can perform detection. Only a weak oxidation current response was generated by the successive addition of AA. All other electrodes showed a higher response compared with CPE.

Starting with EPAA with the –COOH group, the response was 1.76 times higher than that of CPE. Moreover, the corresponding EPI response was 1.57 times higher than that of CPE. This decrease in response may be associated with the absence of the –COOH group. Similarly, S-EPI showed higher sensitivity (1.86 times) than CPE. EPAA had a higher sensitivity compared with EPI (×1.12). The S-EPAA electrode showed the highest response among all with sensitivities, which were 2.45, 1.39, 1.55 and 1.27 times higher than CPE, EPAA, EPI and S-EPI, respectively. The reason is that the sulfonic group on the aniline ring can enhance the resonance effect and increase the attraction between AA and sulfonic acid molecules; this phenomenon enhances the electro-catalytic reaction on the electrode surface of S-EPAA, and the oxidation current response is relatively obvious. The sensitivity of the as-prepared electrodes towards electrochemical sensing of AA is in the order following: S-EPI < EPI < EPAA < S-EPAA.

The linear calibration curve in Figure 5b shows that the change in the peak current and AA concentration had a linear relationship. Table 1 shows the measuring limit of detection, linear dynamic range and sensitivity values for the EPAA, EPI, S-EPI and S-EPAA sensors. The table also summarizes the analytical performance of the other related sensors based on CP-modified electrodes in the present work. A better sensitivity performance was achieved in the present work, compared with the reported PANI systems.

#### 3.3.2. Kinetic Parameter Study

Cyclic voltammograms of the CPEs modified with EPAA, EPI, S-EPAA and S-EPI in PBS towards the oxidation of 2 mM AA, with increasing scan rates between 10 and 500 mV/s, are shown in Figure S5.

**Figure 6:**
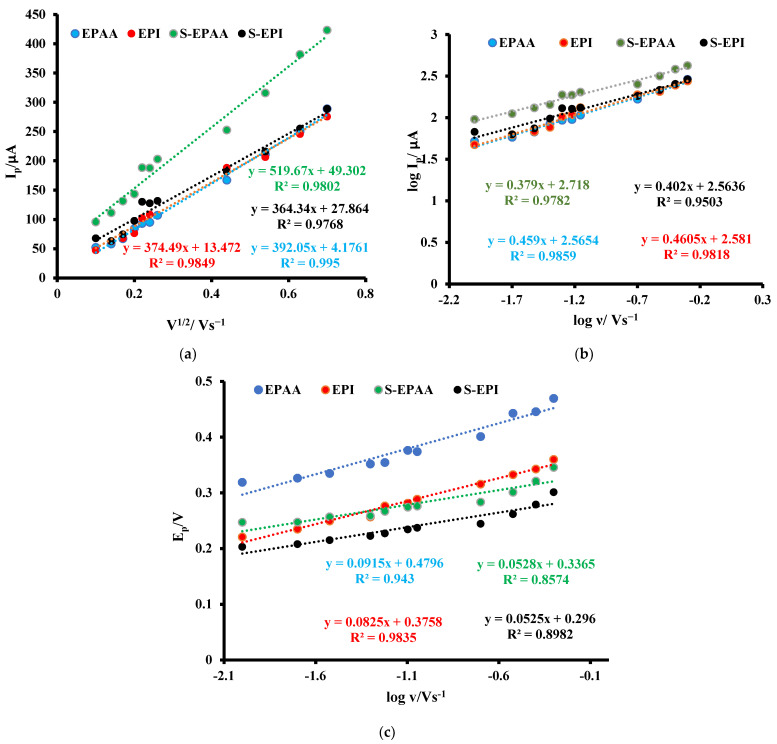
The plot between (**a**) Ip vs. ν^1/2^ (**b**) log Ip vs log ν and (**c**) Ep vs log ν for cyclic voltammograms obtained from EPAA, EPI, S-EPAA and S-EPI in PBS (pH 7) at different scan rates in the presence of 2 mM AA.

The electrode process is diffusion-controlled, as suggested by the linear plots for Ip versus *υ^1/2^* in Figure 6a, and the linear equation for Ip was as follows: 392.05*ν*^1/2^ + 4.1762 (R^2^ = 0.995, EPAA), 374.49*ν*^1/2^ + 13.472 (R^2^ = 0.9849, EPI), 519.67*ν*^1/2^ + 49.302 (R^2^ = 0.9802, S-EPAA) and 364.34*ν*^1/2^ + 27.864 (R^2^ = 0.9668, S-EPI). From the plot of the log ν versus the log Ip (Figure 6b), the linear equations obtained for EPAA, EPI, S-EPAA and S-EPI were 0.459 log *υ* + 2.5654 (R^2^ = 0.9859)*,* 0.4605 log *υ* + 2.581 (R^2^ = 0.9818), 0.379 log *υ* + 2.718 (R^2^ = 0.9782) and 0.402 log *υ* + 2.5636 (R^2^ = 0.9503), respectively. The slopes of 0.459, 0.4605, 0.379 and 0.402 are closer to the theoretical value of 0.5, indicating a purely diffusion-controlled process [48,49,50]. Additionally, the plot between the Ep and log *υ* (Figure 6c) showed good linearity, which indicates that the reaction is irreversible [51]. The regression equations obtained for EPAA, EPI, S-EPAA and S-EPI were Ep = 0.0915 + 0.4796 log *υ* (R^2^ = 0.943), Ep = 0.0825 + 0.3758 log *υ* (R^2^ = 0.9835), Ep = 0.0528 + 0.3365 log *υ* (R^2^ = 0.8574) and Ep = 0.0525 + 0.296 log *υ* (R^2^ = 0.8982), respectively.

#### 3.3.3. Differential Pulse Voltammetry (DPV)

**Figure 7:**
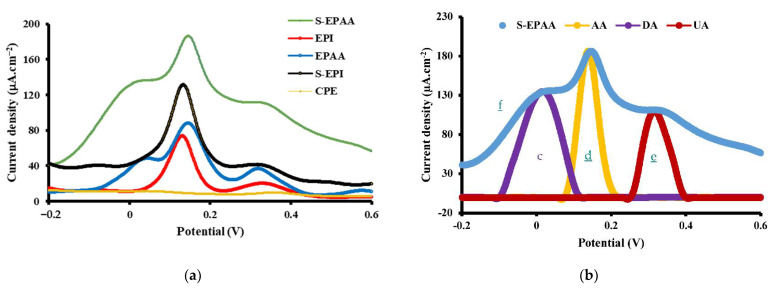
(**a**) DPV obtained with bare CPE, EPAA, EPI, S-EPAA and S-EPI-modified electrodes for a tertiary mixture of 20 µM AA/DA/UA in 0.1 M PBS and (**b**) DPV of 20 µM DA (c), 20 µM AA (d), 20 µM UA (e) and 20 µM AA/DA/UA (f) for the S-EPAA-modified CPE in 0.1 M PBS.

Generally, co-existing electroactive components, such as uric acid (UA) and dopamine (DA), show serious interference in the electrochemical detection of AA. Therefore, the amperometric responses of DA and UA are usually studied by DPV. A tertiary mixture of ascorbic acid (AA), dopamine (DA) and uric acid (UA) was used as an interfering species for the evaluation of selectivity by differential pulse voltammograms (DPV). Figure 7a showed the DPV recorded for bare and modified CPEs in PBS for a mixture of interferents. The concentration of each interferent in a mixture was 20 µM, with a potential window of −0.2 V to 0.6 V. Notably, for the tertiary mixture, bare CPE showed poor selectivity. On the other hand, the selectivity improved with the CPEs modified with EPAA, EPI, S-EPAA and S-EPI, respectively. The CPE modified with S-EPAA exhibited excellent selectivity, with three distinct voltammetric peaks at potentials of 0.022, 0.145 and 0.337 V, corresponding to the oxidation of AA, DA and UA, respectively. The oxidation of DA and UA does not influence the current response of AA, as shown in Figure 7b. This result convincingly indicated that the S-EPAA-modified CPE can be used as a sensor for the selective determination of AA in the presence of interfering components, such as DA and UA. 

## 4. Conclusions

In this work, we reported the preparation of sulfonated and non-sulfonated electroactive monomers (ACAT and S-ACAT) and polymers (EPAA, EPI, S-EPAA, S-EPI), which were applied in electrochemical sensing of ascorbic acid. Based on the electrochemical CV studies, the redox capabilities of the as-synthesized materials were in the following order: S-EPAA > S-EPI > EPAA> EPI. In AA sensing, the CPE modified with S-EPAA had higher sensitivity (172 µM.mM^−^) and relatively low LOD of 0.001 µM at the potential of 0.51 V among all electrodes. Moreover, the S-EPAA electrode exhibited excellent selectivity, with distinct voltammetric peaks. Thus, S-EPAA showed the most reliable results. Keeping in mind the higher sensitivity, good selectivity, low cost and simple preparation, the practical application value of this system will be evaluated in the future using real samples.

## Figures and Tables

**Table 1:** A comparison of various types of ascorbic acid sensors.

Sample	Linear Dynamic Range (µM)	LOD µM	Sensitivity µA·mM^−1^	Reference
AF-MWCNT-EPI/CPE	50–700	4.1	27.5	[33]
EPI4G/CPE	10–1000 mM	18.49	58.56	[34]
EPU/CPE	0.05–0.5	6.1	15.4	[35]
EPA/CPE	50–450	7.1	24.2	[40]
DPSA-doped nanoPANI/SPE	0.5–8 mM	8.3	10.75	[41]
Polyaniline(PANI)/SCPE	30–270	30 ± 3	17.7	[42]
Au-PEDOT/	5–300	2.5	0.875	[43]
PANIS/Au/GCE	80–200	0.08		[44]
Gold decorated SiO_2_@PANI core-shell microspheres	0.15–8	3.775	21.8	[45]
EMGON5-1		1.54	78.63	[46]
AF-MWCNT/EPU/CPE	0.01–0.91	1.2	35.3	[47]
EPI	0.02–0.3 mM	28	127	This work
S-EPI	0.02–0.3 mM	10	134	This work
EPAA	0.02–0.3 mM	10	127	This work
S-EPAA	0.02–0.3 mM	1	172.23	This work

## Data Availability

The data presented in this study are available on request from the corresponding author.

## Supplementary Materials

The following supporting information can be downloaded at: https://www.mdpi.com/article/10.3390/polym14173487/s1, Figure S1: The representative characterization of ACAT (a) FTIR (B) Ion-trap mass spectrum and (c) H-NMR; Figure S2: The representative characterization of S-ACAT (a) FTIR (B) Ion-trap mass spectrum. Figure S3: Cyclic voltammograms of blank ITO, ACAT and S-ACAT, Figure S4: Cyclic voltammograms obtained of EPAA (a), EPI (b), S-EPAA (c) and S-EPI (d) in at different concentration of 0, 2, 4, 6, 8 and 10 mM of AA in PBS. Figure S5: Cyclic voltammograms obtained of EPAA (a), EPI (b), S-EPAA (c) and S-EPI (d) in PBS (pH ~ 7) at different scan rates in the presence of 2 Mm AA.
