# Supplementary material for: Comparative Studies on Carbon Paste Electrode Modified with Electroactive Polyamic Acid and Corresponding Polyimide without/with Attached Sulfonated Group for Electrochemical Sensing of Ascorbic Acid
Source: Polymers (Basel). 2022 Aug 25;14(17):3487. doi: 10.3390/polym14173487 (PMC9460801; doi:10.3390/polym14173487)
Supplement: Supplementary file 1 [file polymers-14-03487-s001.zip › polymers-1878032-supplementary.pdf]

Synthesis of N,N′-bis(4′-aminophenyl)−1,4-quinonenediimine (ACAT)

ACAT was synthesized by the procedure reported in the literature as shown in scheme 1.

Preparation of 3,6-bis((4-aminophenyl)imino)cyclohexa-1,4-diene-1-slfonic acid (S-ACAT)

Add 3.0 g of 2,5-diaminobenzenesulfonated into solution of 80mL of 95% EtOH and 200 mL of HCl and stir well, then put inside ice bath and for stir 10 minutes. Add the Ammonium persulfate, followed by 3.0 g of aniline and stir for 5 min. and then remove from the ice bath. The product was filtered and washed with a mixture of 160mL isopropanol and 60mL HCl. Wait for the filtrate to dry, then remove the product from the filter paper and soak it in 1M NH_4OH 100mL for one hour. Take 700mL of isopropanol and force the solution in the previous step to precipitate in it, stirring for 24 hours. After the filtrate is dried, rinse and dry with 50 mL of isopropanol and put it into a vacuum oven and dry it at 60°C for two hours, then grind it for use. The S-ACAT yield was approximately 47%.

a)

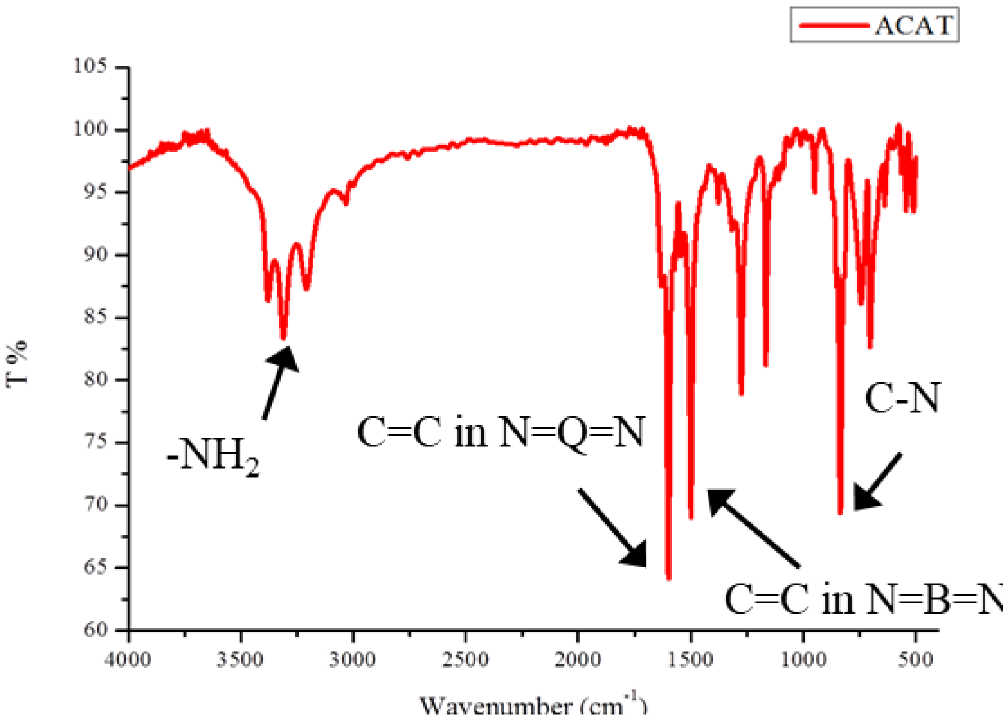

b)

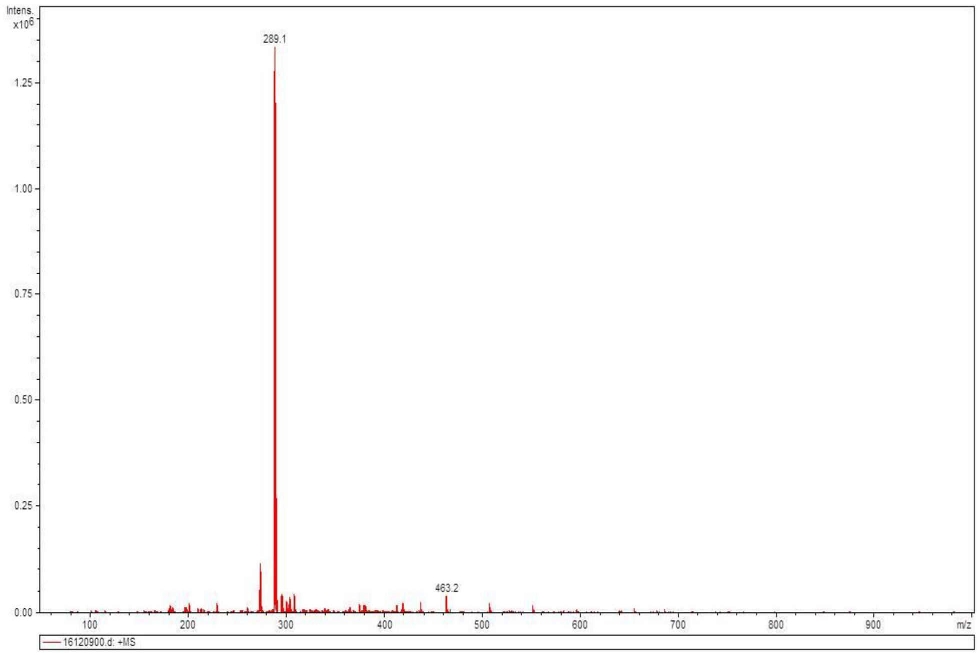

Fig. S1 The representative characterization of ACAT (a) FTIR (B) Ion-trap mass spectrum and (c) H-NMR

a)

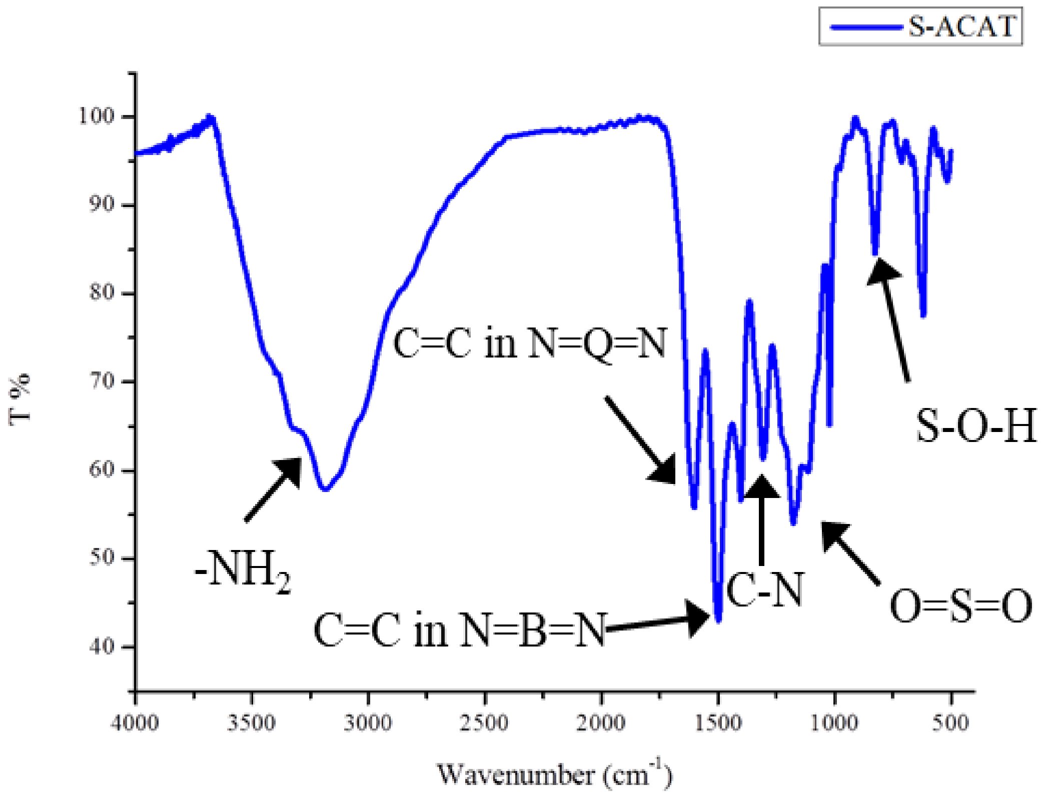

b)

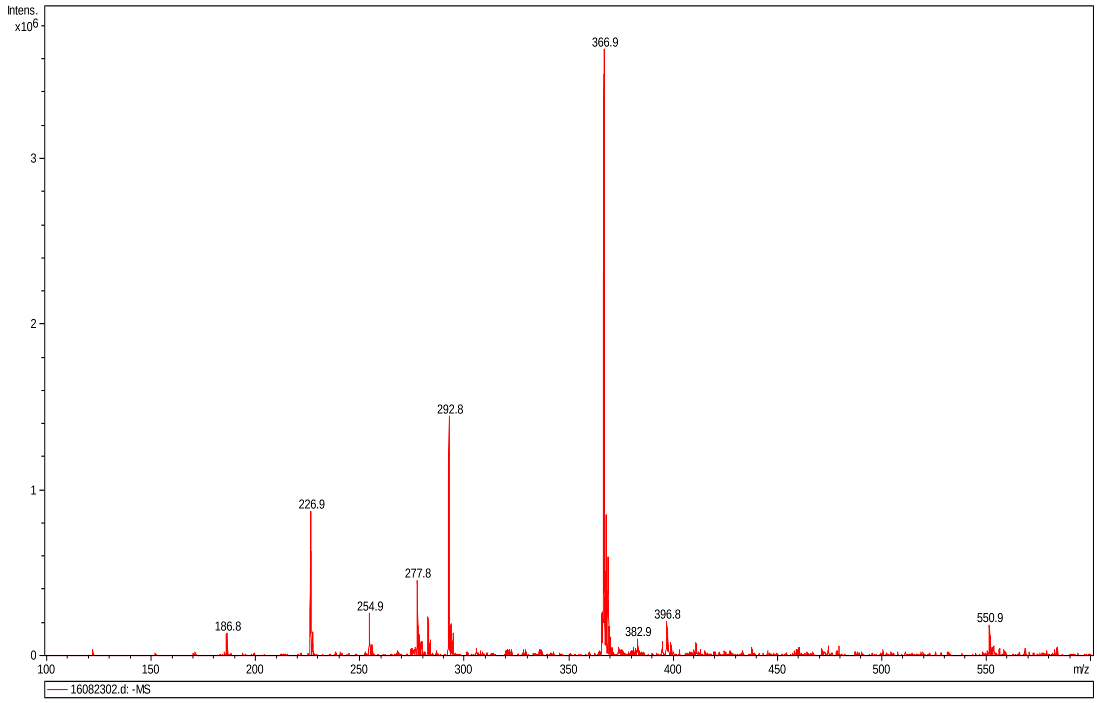

Fig. S2. The representative characterization of S-ACAT (a) FTIR (B) Ion-trap mass spectrum.

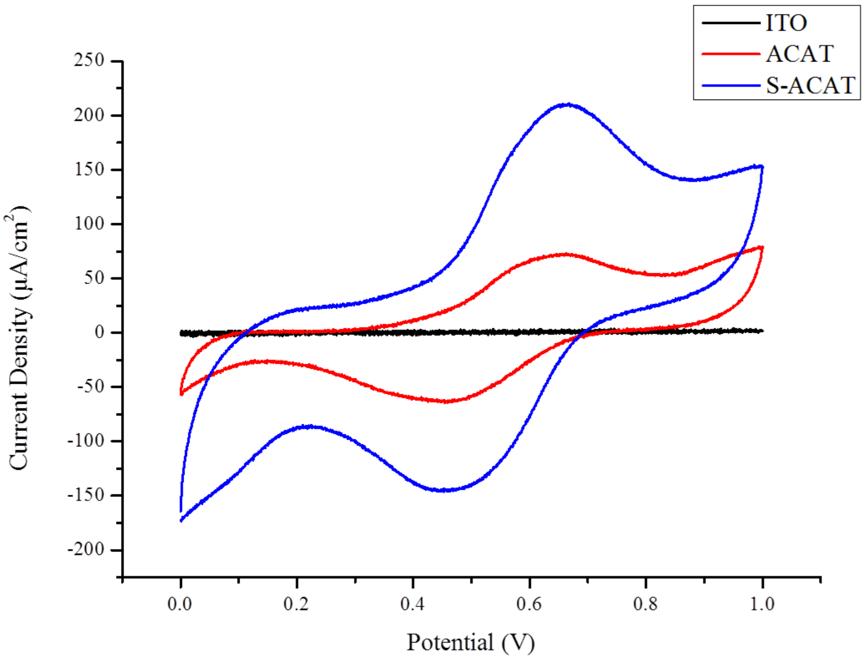

Fig. S3 Cyclic voltammograms of blank ITO, ACAT and S-ACAT.

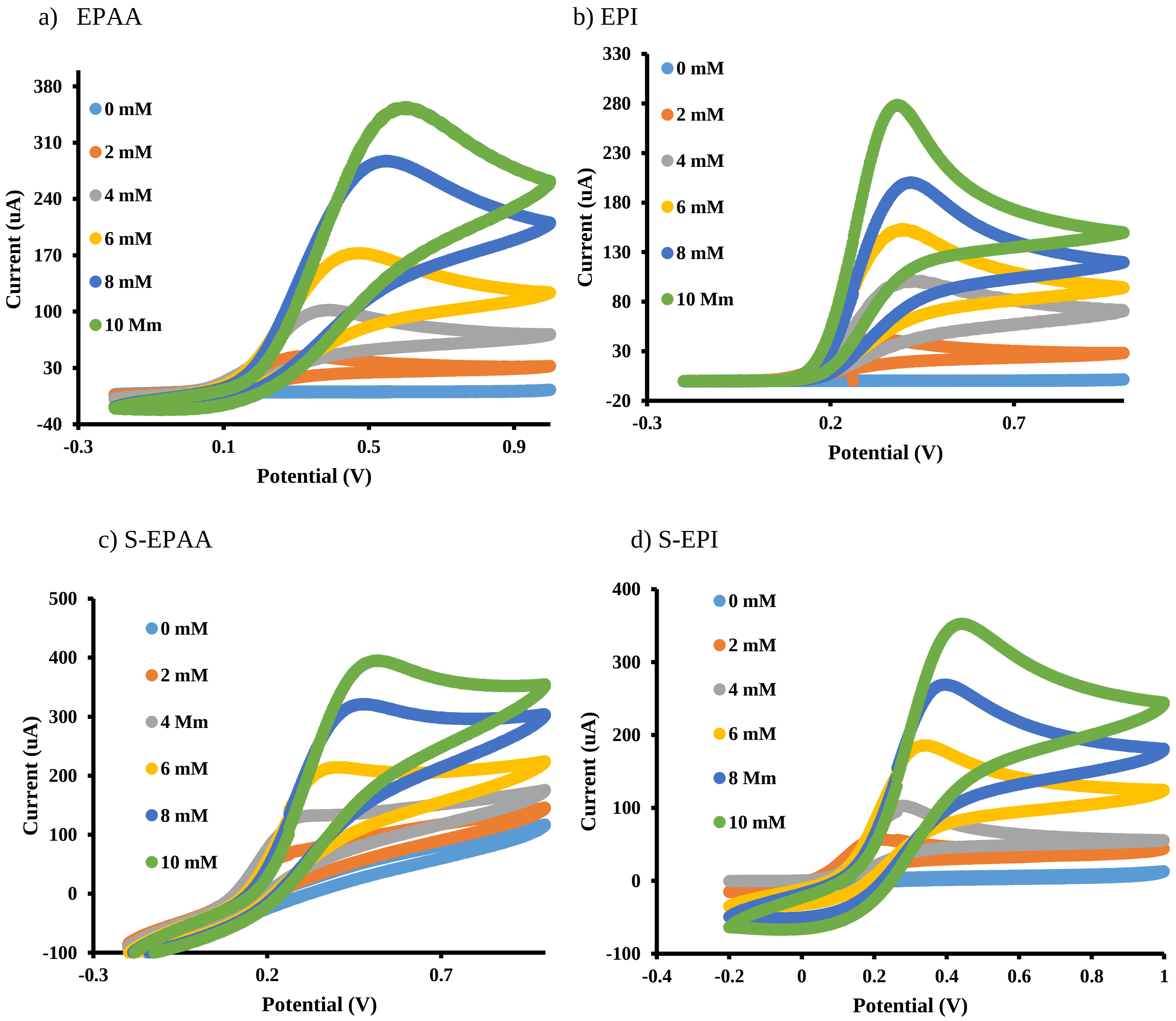

Fig. S4. Cyclic voltammograms obtained of EPAA (a), EPI (b), S-EPAA (c) and S-EPI (d) in at different concentration of 0, 2, 4, 6, 8 and 10 mM of AA in PBS.

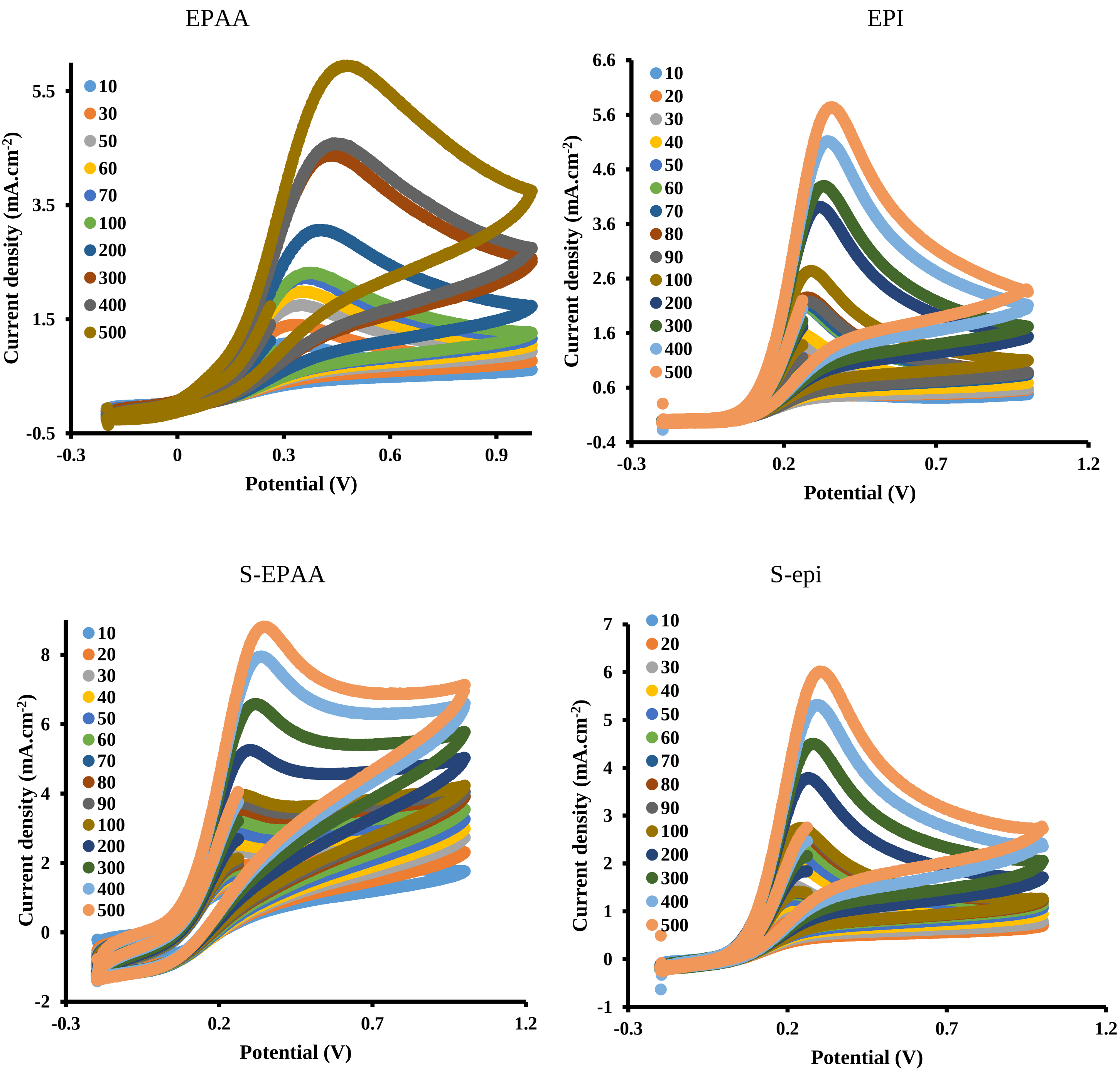

Fig. S5. Cyclic voltammograms obtained of EPAA (a), EPI (b), S-EPAA (c) and S-EPI (d) in PBS (pH ~ 7) at different scan rates in the presence of 2 Mm AA.
